# Supplementary material for: SMARTcleaner: identify and clean off-target signals in SMART ChIP-seq analysis
Source: BMC Bioinformatics. 2018 Dec 27;19:544. doi: 10.1186/s12859-018-2577-4 (PMC6307164; doi:10.1186/s12859-018-2577-4)
Supplement: Supplementary file 2 — Figure S1. Examples of pileup of 2nd reads of ChIP-seq read pairs near consecutive and interrupted poly(T/A) sites. Figure S2. Numbers of ChIP-seq fragments mapped to genomic regions with consecutive and interrupted poly(T/A) sequences. Figure S3. Cleaning results of Dataset 2. Figure S4. Examples of the four groups of Olig2 ChIP-seq peaks. Figure S5. Cartoons illustrating the alignment of paired-end reads to poly(T/A) regions. Figure S6. Cartoons illustrating read alignment near genomic poly(T/A) sites. Figure S7. Determination of the window size of regions containing the false reads at (a) poly(T) or (b) poly(A) sites. Figure S8. Resampling strategy at the genomic poly(T/A) sites. (PDF 498 kb) [file 12859_2018_2577_MOESM2_ESM.pdf]

Additional file 2

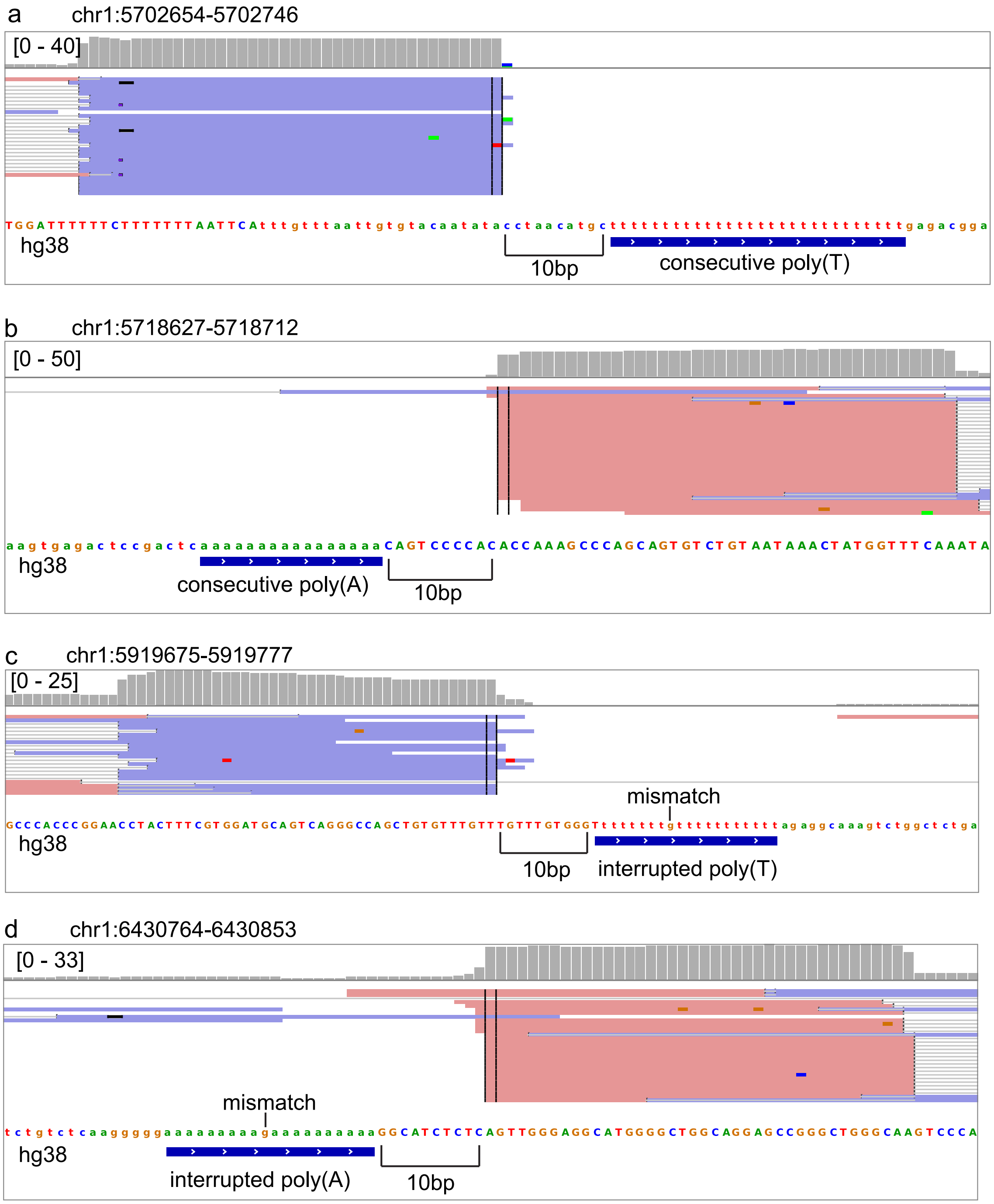

Figure S1. Examples of pileup of 2nd reads of ChIP-seq read pairs near consecutive and interrupted poly(T/A) sites. The authors trimmed the 2nd reads during data submission to the GEO database (SRR3229031), resulting in a 10 bp gap between the edge of poly(T/A) sites and the 2nd reads. The images were exported from Integrative Genomics Viewer (IGV) as SVG files and edited in Adobe Illustrator.

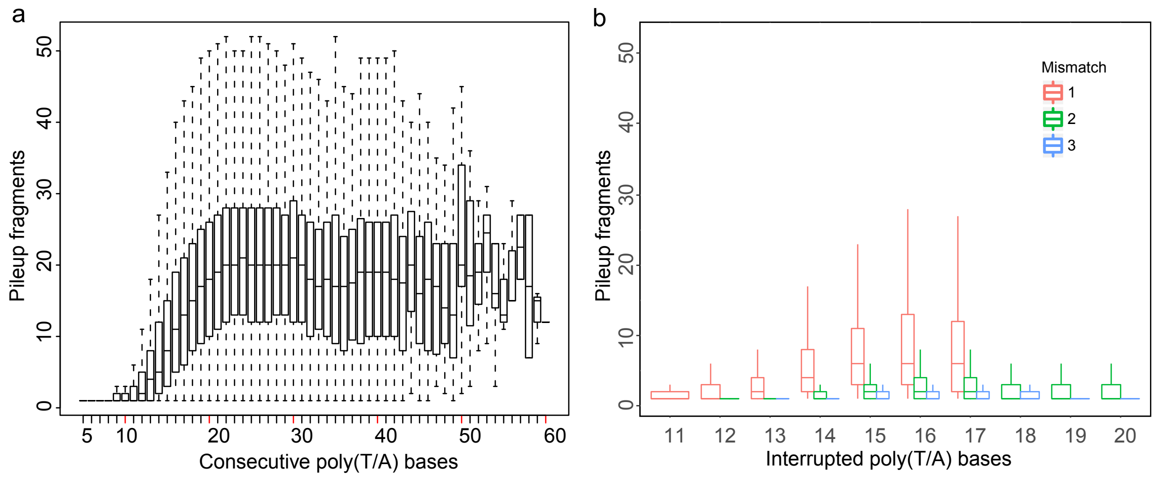

Figure S2. Numbers of ChIP-seq fragments mapped to genomic regions with consecutive and interrupted poly(T/A) sequences. PE sequencing data for SRR3229031 (Dataset 1 in Additional file 1: Table S1) was used and only genomic regions with reads were analyzed.

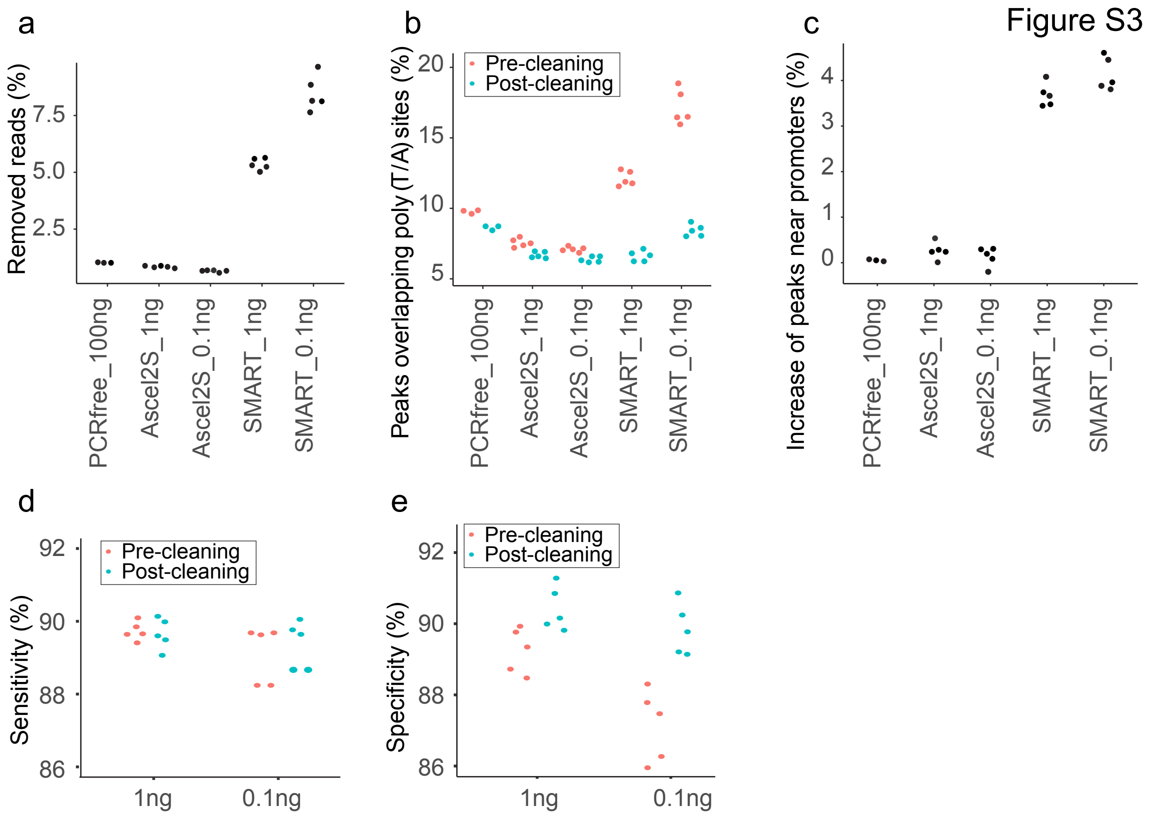

Figure S3. Cleaning results of Dataset 2. **a**, Percentages of removed reads in control and SMART-based datasets. **b**, Overlaps of peaks with genomic poly(T/A) sites. **c**, Increase of post-cleaning peaks at promoter regions. **d**, Sensitivity (% PCR-free reference peaks detected). **e**, Specificity (% peaks found in PCR-free reference peaks).

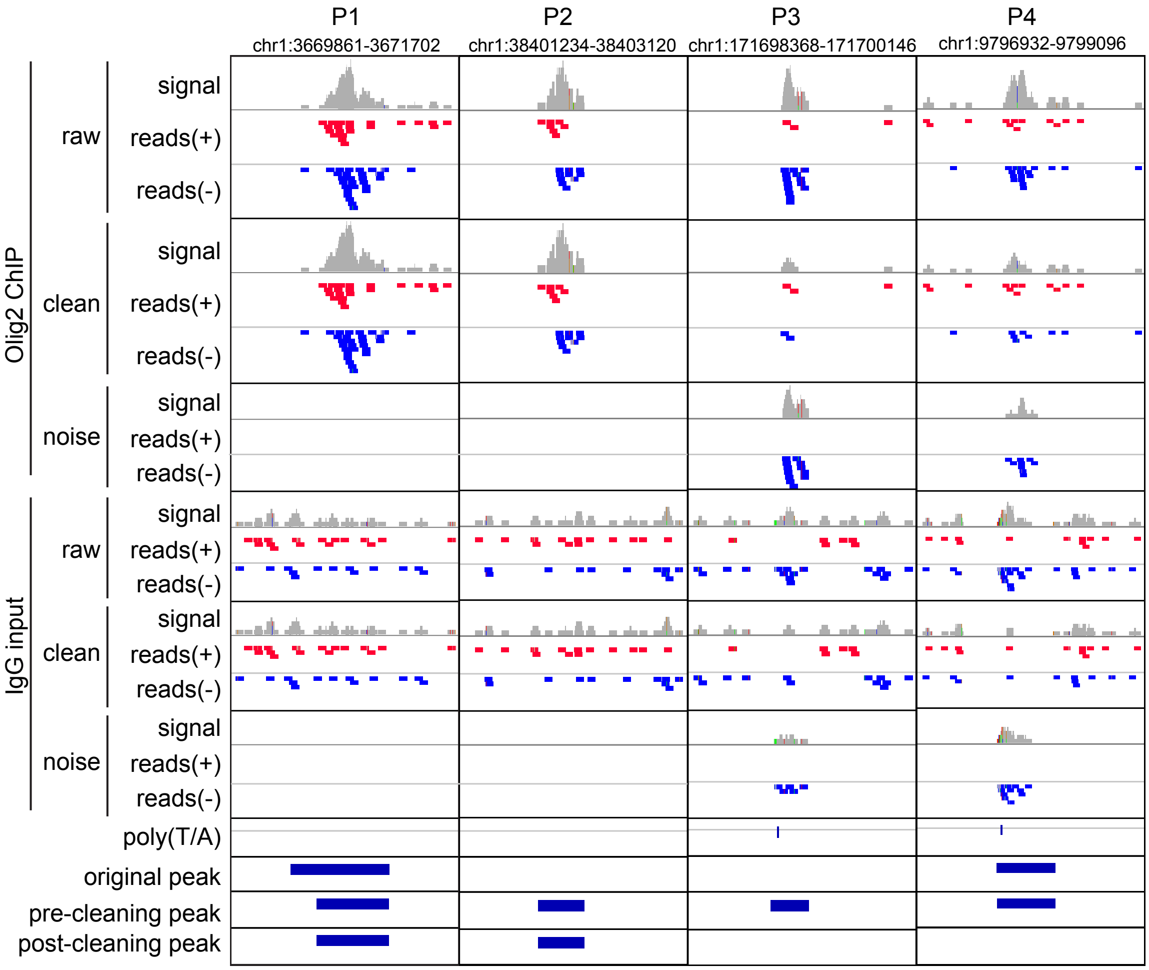

Figure S4. Examples of the four groups of Olig2 ChIP-seq peaks. The four columns indicate the four groups of peaks: P1, P2, P3, and P4. The rows show the reads on +/- strands in raw, clean, and noise bam files for ChIP and IgG control samples. The poly(T/A) sites on the reference genome (mm10) and peak locations called in the original study, pre-cleaning data, and post-cleaning data were showed in the last four rows.

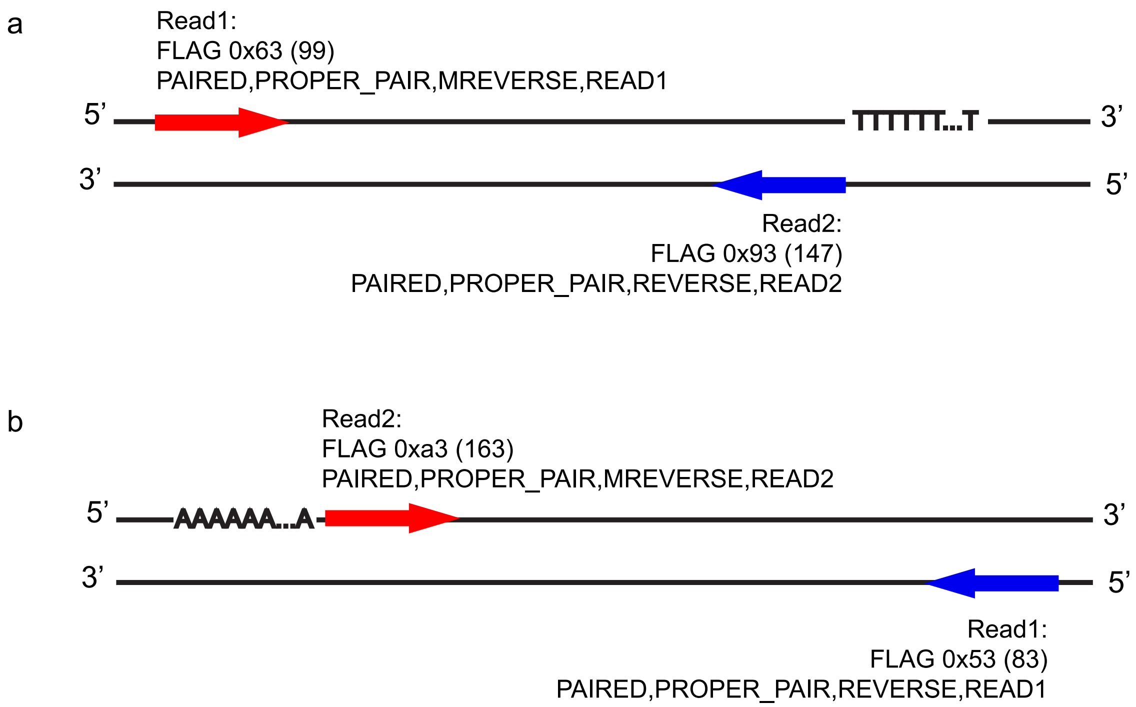

Figure S5. Cartoons illustrating the alignment of paired-end reads to poly(T/A) regions. SMARTcleaner uses "FLAG" information in the 2nd column of SAM/BAM files to tell the identity of a read. Note that the FLAG of a read does not have to be identical to what is shown here, as long as the alignment software set the required bits in the FLAG field.

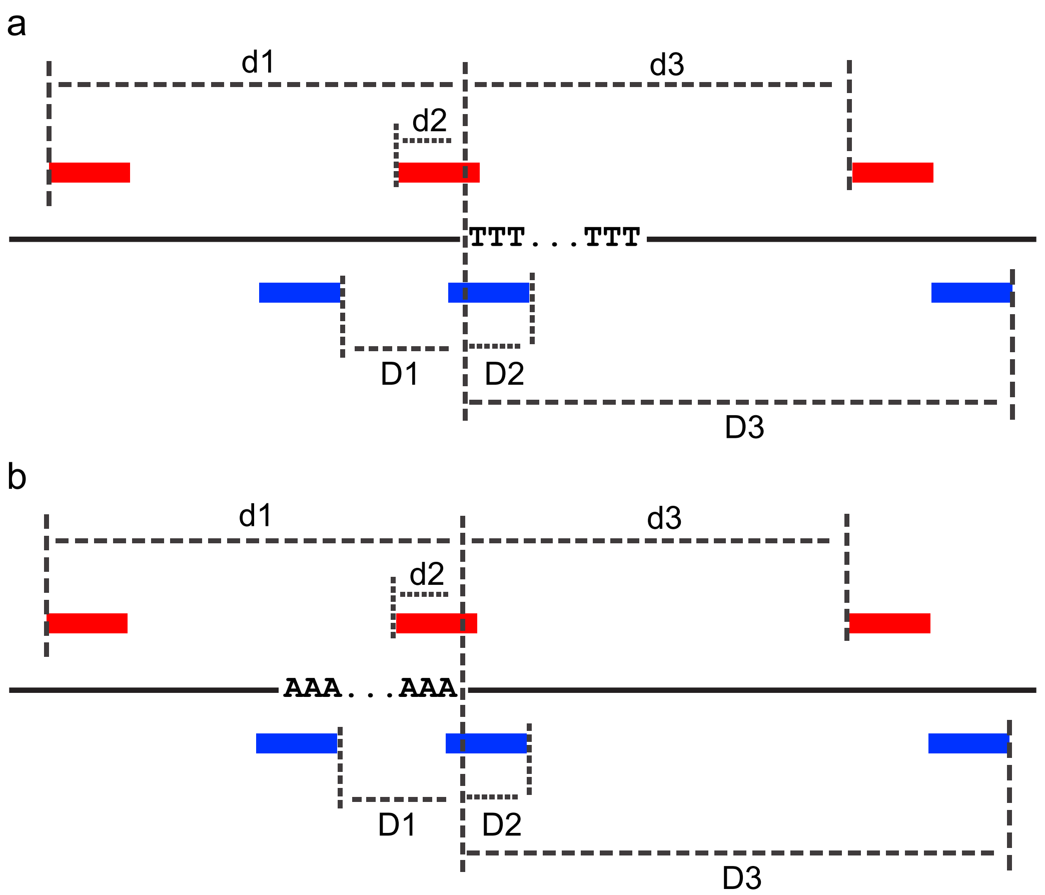

Figure S6. Cartoons illustrating read alignment near genomic poly(T/A) sites. Reads mapped to "+" and "-" strand were in red and blue, respectively. For reads on "+" strand, the distance (d1, d2 or d3) is calculated from the left ends of the three reads to the left ends of poly(T) (**a**) or the right ends of poly(A) (**b**). For reads on "-" strand, the distance (D1, D2 or D3) is calculated from the right ends of the three reads to the left ends of poly(T) (**a**) or the right ends of poly(A) (**b**).

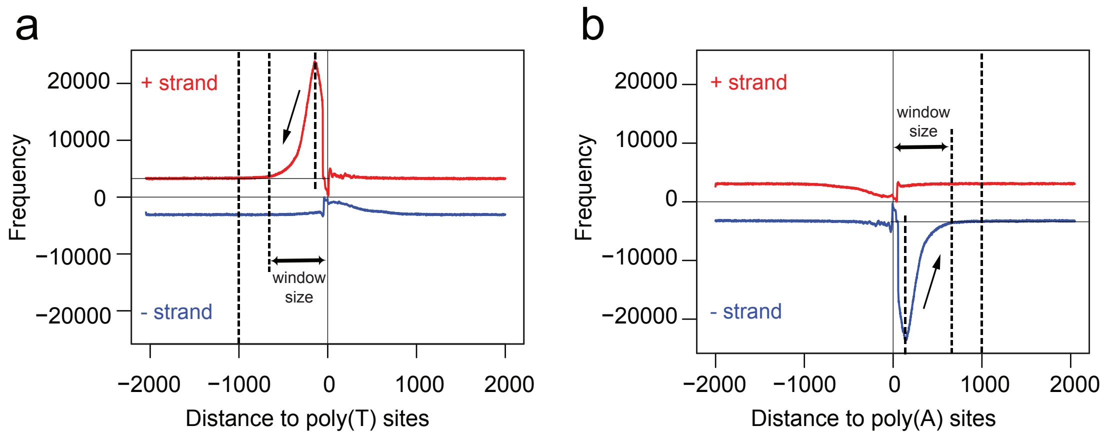

Figure S7. **Determination of the window size of regions containing the false reads at (a) poly(T) or (b) poly(A) sites**. The frequency distribution of the distances for ChIP-seq fragments to the poly(T/A) sites is computed at the whole

genome level. An enrichment near poly(T/A) sites is expected if false priming has occurred and the enrichment peak is expected to be about 1kb, since the fragment lengths are usually <1 kb in current ChIP-seq experiments. We thus use 1-2kb near poly(T/A) sites as baseline and check the frequency of distances in the 0-1kb near poly(T/A) sites. Starting from the highest value of the distance frequency, we search for the distance point where the value is reduced to the max baseline value and then use the distance as the window size. The larger number is used if the two numbers for poly(T) and poly(A) are different.

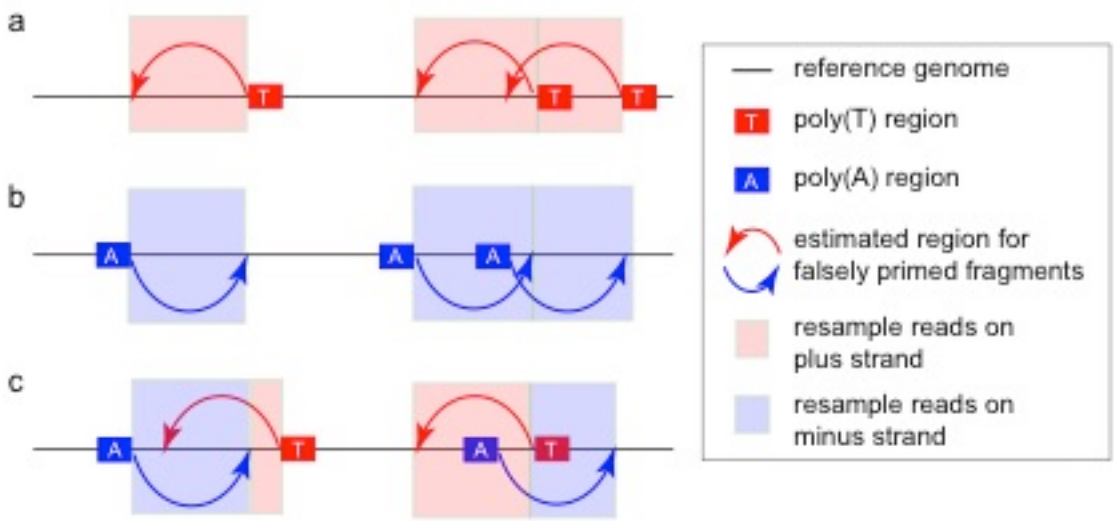

Figure S8. Resampling strategy at the genomic poly(T/A) sites. The resampling regions are located at the upstream of poly(T) sites (**a**) or the downstream of poly(A) sites (**b**). In the events that a poly(T) and a poly(A) region overlap (**c**), sampling of reads in the overlapping region will be in the order of the appearance of poly(T) and poly(A) in the reference genome.
